# Exploring the Utility of ChatGPT in Cleft Lip Repair Education

**DOI:** 10.3390/jcm14030993

**Published:** 2025-02-04

**Authors:** Monali Mahedia, Rachel N. Rohrich, Kaiser O’Sahil Sadiq, Lauren Bailey, Lucas M. Harrison, Rami R. Hallac

**Affiliations:** 1 Department of Surgery, Rutgers University—NJMS, Newark, NJ 07103, USA; 2 Department of Plastic and Reconstructive Surgery, MedStar Georgetown University Hospital, Washington, DC 20007, USA; 3 Department of Surgery, University of Washington, Seattle, WA 98195, USA; 4 Department of Plastic Surgery, University of Texas Southwestern Medical Center, Dallas, TX 75390, USA; 5 Analytical Imaging and Modeling Center, Children’s Health, Dallas, TX 75235, USA

**Keywords:** cleft lip repair, patient education, large language model, ChatGPT, generative artificial intelligence, healthcare information quality

## Abstract

**Background/Objectives**: The evolving capabilities of large language models, such as generative pre-trained transformers (ChatGPT), offer new avenues for disseminating health information online. These models, trained on extensive datasets, are designed to deliver customized responses to user queries. However, as these outputs are unsupervised, understanding their quality and accuracy is essential to gauge their reliability for potential applications in healthcare. This study evaluates responses generated by ChatGPT addressing common patient concerns and questions about cleft lip repair. **Methods**: Ten commonly asked questions about cleft lip repair procedures were selected from the American Society of Plastic Surgeons’ patient information resources. These questions were input as ChatGPT prompts and five board-certified plastic surgeons assessed the generated responses on quality of content, clarity, relevance, and trustworthiness, using a 4-point Likert scale. Readability was evaluated using the Flesch reading ease score (FRES) and the Flesch–Kincaid grade level (FKGL). **Results**: ChatGPT responses scored an aggregated mean rating of 2.9 out of 4 across all evaluation criteria. Clarity and content quality received the highest ratings (3.1 ± 0.6), while trustworthiness had the lowest rating (2.7 ± 0.6). Readability metrics revealed a mean FRES of 44.35 and a FKGL of 10.87, corresponding to approximately a 10th-grade literacy standard. None of the responses contained grossly inaccurate or potentially harmful medical information but lacked citations. **Conclusions**: ChatGPT demonstrates potential as a supplementary tool for patient education in cleft lip management by delivering generally accurate, relevant, and understandable information. Despite the value that AI-powered tools can provide to clinicians and patients, the lack of human oversight underscores the importance of user awareness regarding its limitations.

## 1. Introduction

Generative artificial intelligence (AI) tools, such as the chat generative pre-trained transformer (ChatGPT; OpenAI, USA), have gained substantial popularity, driven by their advanced language processing capabilities and potential to transform fields like healthcare and education. These tools represent a category of large language models (LLMs), which are advanced applications specializing in natural language processing, identifying relationships between words and phrases within user input [1,2]. Rapid advancements in AI architecture and neural networks have broadened the applicability of these models across various domains, enabling them to interpret inquiries and deliver tailored responses [3]. Current versions of ChatGPT are trained on extensive text datasets including medical and educational materials and are freely accessible to the general population with additional functionality available to monthly subscribers [4]. ChatGPT remains at the forefront of LLM development, noted for generating detailed, complex responses that have rendered it a valuable resource for healthcare professionals and patients alike [5,6].

Cleft lip and palate are among the most common congenital craniofacial anomalies, affecting approximately 1 in 700 live births worldwide [7,8]. These conditions present unique challenges for both patients and healthcare providers, as they often require a multidisciplinary approach involving surgical repair, orthodontics, speech therapy, and psychological support [9,10]. Each stage of care carries its own complexities, from surgical planning to long-term functional and aesthetic outcomes, making comprehensive patient education a vital component of effective treatment [11]. Families must navigate intricate medical terminology, understand potential risks and benefits, and align their expectations with realistic outcomes, all of which underscore the importance of accessible and accurate educational resources.

Patient education and understanding are critical in procedures such as cleft lip repair, where decisions often have long-lasting implications for quality of life. Beyond the clinical setting, patients and families increasingly rely on online health resources for information, making the internet an important resource for health education [12]. These resources shape patient perceptions and behaviors, which can in turn influence expectations and patient satisfaction [13,14].

While online health information varies in quality and accuracy, AI-generated content has the potential to further enhance patient education. As AI-generated medical information becomes increasingly accessible, evaluating the quality and accuracy of generated information is essential to determine its value and limitation.

Literature on the application of AI and LLMs in craniofacial and pediatric surgery remains sparse, with preliminary studies demonstrating the potential to support research and medical training [15,16]. Other specialties, including dermatology and otorhinolaryngology, have explored LLMs for educational applications, yielding promising results in enhancing medical knowledge dissemination and patient understanding [17,18,19,20].

This study aims to evaluate the quality, accuracy, and readability of ChatGPT-generated responses to common patient questions and concerns related to cleft lip repair to determine its potential effectiveness as a resource for patient education. This study focuses on ChatGPT’s capacity to provide clear, reliable, and accessible information that improves patient understanding and informs decision-making in the context of cleft lip repair. We seek to determine its potential role as a supplementary tool for improving patient understanding while identifying its limitations in clinical communication.

## 2. Materials and Methods

### 2.1. Study Design and ChatGPT

This study employed a cross-sectional evaluation of the quality of patient education material generated by ChatGPT-4 concerning cleft lip and its repair. ChatGPT (OpenAI, CA, USA), is a widely accessible LLM available at minimal to no cost. Text inputs serve as prompts for responses generated in real-time, with output varying in content, style, and length depending on the prompt and saved user history.

In this study, each prompt was entered as a new, standalone conversation to prevent the model from using prior inputs, ensuring that each response reflected ChatGPT’s baseline or “standard” output. This approach simulated typical user interactions and ensured reproducibility in evaluating responses. Prompt engineering techniques were intentionally omitted, as these methods may not reflect typical user inputs and could bias results [21]. ChatGPT was allowed to generate responses without specifying format, length, or quality restrictions to simulate common user behavior. This setup aimed to mimic how patients or caregivers might use the tool without prior technical expertise. All responses were collected in a single, controlled session to maintain consistency.

### 2.2. Assessment and Prompts

The patient information page on cleft lip provided by the American Society of Plastic Surgeons (ASPS) was used to develop ten question prompts for this study [22]. These questions addressed information deemed most relevant to patients and caregivers considering cleft lip repair, providing a comprehensive foundation for informed decision-making (Table 1).

The questions were designed to assess a range of informational needs, including medical explanations, procedural details, recovery expectations, risk factors, and the multidisciplinary nature of cleft care. This ensured that the evaluation covered all critical aspects of patient education.

ChatGPT’s responses were evaluated using a 4-point Likert scale across four dimensions: content accuracy, clarity, relevance, and trustworthiness, as well as overall quality, with scores ranging from 1 (lowest) to 4 (highest) (Table 2). Five board-certified plastic surgeons were recruited to assess each response based on these criteria, drawing on their clinical expertise to rate the outputs. Additionally, readability metrics were calculated for each response to assess their accessibility to a general audience. The surgeons were also asked to evaluate the potential usefulness of AI-generated responses in clinical practice, providing open-ended feedback detailing whether they would consider incorporating such tools as part of patient education and engagement.

### 2.3. Readability

Readability metrics were calculated using the Flesch reading ease score (FRES) and Flesch overall quality, Kincaid grade level (FKGL) based on the sentence and word counts of ChatGPT responses [19,23,24]. These metrics offer additional perspective on the accessibility and clarity of AI-generated content, particularly for audiences unfamiliar with medical literature. Given that patient education materials are recommended to target a literacy level around the sixth grade, the readability scores were compared against this preset standard.

## 3. Results

All responses were generated and recorded in September 2024 (Appendix A). Five board-certified plastic surgeons assessed the ten responses in full. No responses resulted in errors or required regeneration.

**Figure 1:**
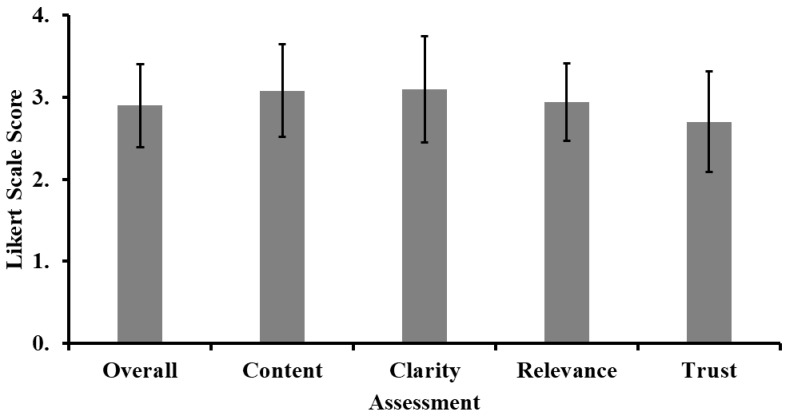
Aggregated assessment outcomes for each domain of ChatGPT responses, error bars representing the standard deviation of responses.

Overall, the assessments were satisfactory, with an aggregated mean rating of 2.9 on the Likert scale Figure 1). Clarity and content quality received the highest ratings, each scoring 3.1 ± 0.6, while trustworthiness had the lowest rating at 2.7 ± 0.6.

**Figure 2:**
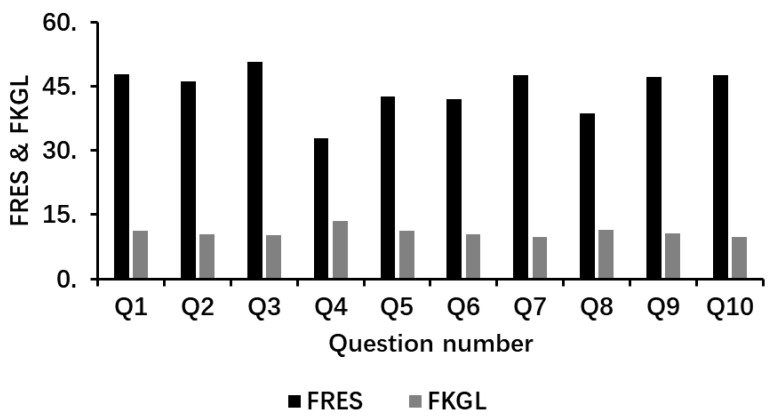
The Flesch reading ease score (FRES; Black) and Flesch–Kincaid grade level (FKGL; Grey) metrics for each of the ten questions.

Readability metrics revealed a mean FRES of 44.35 and an FKGL of 10.87, indicating a reading level above the recommended sixth-grade target (Figure 2). The average output length across the ten prompts was 302 ± 100 words, with variations corresponding to the scope and specificity of each question. These results provide quantitative insights into the linguistic characteristics and verbosity of the generated outputs.

None of the responses achieved a full score from all assessors in any category. Additionally, responses lacked references to verified sources, books, or websites that patients could consult to verify the veracity of provided information. When asked about the potential integration of AI tools in clinical practice, only one out of five clinicians responded affirmatively, while the others were ambivalent.

The LLM responses addressed key concerns such as scarring, facial symmetry, and speech outcomes (Q8), offering useful introductory information but lacking the depth of detailed clinical consultations. In response to Q9, the LLM correctly emphasized the importance of a multidisciplinary team, receiving unanimous satisfactory ratings. For Q10, practical guidance was provided on preparing for cleft lip surgery, offering valuable support for parents. While relevant and informative, the responses lacked detail to fully address all potential questions, highlighting ChatGPT’s role as a supplementary resource to fill information gaps.

## 4. Discussion

### 4.1. Key Findings

Traditionally, patient education has been delivered exclusively by clinicians, but the emergence of the internet and advancing technologies has expanded the range of accessible health information. AI-generated medical content represents a new avenue for patient education that warrants thorough evaluation. This study aimed to assess the quality of ChatGPT-generated responses for individuals seeking information about cleft lip repair. The findings indicate that ChatGPT provided responses of satisfactory quality, without gross inaccuracies that could lead to potential harm. Notably, the responses were generally well-constructed and avoided using complex technical terms, making them accessible to a broader audience.

### 4.2. Comparison of Current Educational Resources for Cleft Lip

A previous evaluation of the quality of cleft lip educational resources revealed that even professionally produced content intended for patient use often contained only basic educational material [25]. A significant proportion of assessed websites lacked critical perioperative instructions (~80%) and diagnostic details (~65%). While these deficiencies may not align with the patients and caregivers’ priorities, they represent areas for improvement. Webpages indexed by search engines, including those authored by non-medical professionals, were found to be of even lower quality [26]. This assessment applied a validated tool for patient information: the modified Ensuring Quality Information for Patients (EQIP) instrument, which measures quality, bias, reliability, and clarity. Out of a maximum score of 36, the median score was 19, reflecting poor quality—similar to findings for other facial procedures with limited explanations [14,27]. However, academic centers and hospitals produced webpages that scored higher (>22) compared to the cohort average, suggesting they deliver better, though still insufficient, information [25,26]. Notably, medical practitioners, including plastic, oral, and maxillofacial surgeons, produced websites with lower scores (<22) that often omitted discussions on risks and complications.

Other formats, such as videos on cleft lip repair and surgery, demonstrated only moderate educational quality [28]. Both videos and textual materials share similar limitations in delivering comprehensive and reliable content about the pathology and procedure of interest. Collectively, these studies highlight multiple areas requiring improvement in existing patient education materials for cleft lip, particularly including perioperative information and risk discussions.

### 4.3. Explanations of Findings

The combined functional and aesthetic implications of cleft lip repair on quality of life produce concerns that may differ from those of other surgical procedures [29,30]. The primary concerns include the social and psychological impacts on children. The responses generated by the LLM addressed many of these issues, such as scarring, facial symmetry, and speech outcomes (Q8). While these responses can be helpful as an introductory discussion, they lack the depth necessary to replace detailed clinical consultations entirely. The LLM appropriately emphasized that healthcare teams should further elaborate on potential risks and complications during consultations and follow-up visits. Notably, in response to Q9, the LLM correctly identified that a multidisciplinary team is essential for comprehensive care, which received unanimous satisfactory ratings. While these responses provided key information comparable to a surgeon’s input, they lacked sufficient detail to preempt additional questions or concerns.

Practical guidance was also included in responses to Q10, which addressed steps parents might consider when preparing for their child’s cleft lip surgery. This type of content is particularly valuable for parents who may be anxious or unfamiliar with procedural logistics. Overall, the LLM outputs for these prompts were relevant and addressed key concerns, incorporating specific references to perioperative information and risks often overlooked in typical web-based content. Thus, ChatGPT can fill certain information gaps, supplementing available resources.

It is important to note that the assessed responses were generated using specific, well-defined prompts outlined by the ASPS [22]. These questions were selected based on their importance for patient and caregiver participation in shared decision-making. Given the structured nature of the prompts, it is not surprising that ChatGPT produced reasonable responses. However, patients or parents may face difficulty in adequately phrasing prompts to elicit such targeted responses. Further research is needed to explore the differences between patient-driven queries and the critical information the multidisciplinary team prioritized for informed decision-making.

### 4.4. Implications and Recommended Actions

AI, particularly machine learning (ML) and deep learning (DL), has proven valuable in plastic surgery for assessing various conditions, including ear deformities [31,32,33], craniosynostosis [34,35,36], and cleft lip and palate [37,38]. With the rapid advancement of large language models (LLMs), research has increasingly explored their potential applications in plastic surgery education and patient engagement [39]. Gómez-Cabello et al. examined the role of LLMs in intraoperative decision support, comparing the performance of ChatGPT-4 and Gemini [40]. Their study found that while ChatGPT-4 generated more precise and relevant responses, both models showed promise as intraoperative support tools. However, inconsistencies across different procedures highlight the need for further model refinement to enhance reliability in surgical settings. Similarly, Mohapatra et al. explored the integration of LLMs into plastic surgery residency training, suggesting that these models could enhance learning by providing interactive, personalized educational experiences [41]. Their findings position LLMs as valuable adjuncts for future surgical education.

Shared decision-making is crucial in fostering trust and satisfaction between patients and clinicians [42,43]. Effective medical education facilitated through clinician guidance as well as self-directed learning impacts patients’ health-seeking behaviors and comprehensive perioperative information can reduce preoperative anxiety and enhance patient experience [44]. Inadequate communication has often been linked to patient dissatisfaction and adverse outcomes [45], which may be addressed through supplemental educational information provided by LLMs. ChatGPT’s strength lies in its natural language processing capabilities, enabling it to convey concise and clear medical information in a structured manner. This capability also positions it as a tool that can enhance professionally produced content, making it more patient-friendly by summarizing and rephrasing medical information using more straightforward language for improved readability [2,18,19,46]. This potential is supported by readability metrics, which indicate an average literacy level around the 10th grade—consistent with its performance observed in other facial procedure contexts.

Enhanced communication through LLMs can also mitigate the risk of malpractice claims by aligning patient expectations with realistic postoperative outcomes [47,48,49]. Although AI tools can aid clinicians with textual communication, current models cannot generate relevant, high-quality clinical images. Surgical procedures, especially those where aesthetic outcomes are emphasized, may be easier to understand with visual aids, thus integration of AI image generation may further enhance the capacity to develop comprehensive patient educational materials.

A notable concern is the documented occurrence of AI hallucinations, where LLMs generate inaccurate information when unable to respond adequately [1,50]. While the responses in this study did not include overtly incorrect information, this remains a risk with unsupervised use, as patients acting on unreliable advice could face serious consequences. Continued evaluation of AI tools is essential to understand their role in patient education and consent processes. Similar to other web-based and video content, ChatGPT does not provide references or source verification, underscoring the need for cautious application. AI should not replace professional communication, as misinformation poses significant risks to patient safety and exposes clinicians to potential liability [48,51].

### 4.5. Strengths and Limitations

This study provides an initial evaluation of ChatGPT’s ability to generate patient education content for cleft lip repair. A key strength is the use of standardized patient education materials from the American Society of Plastic Surgeons (ASPS) to formulate representative questions, ensuring clinical relevance. Additionally, responses were evaluated by multiple board-certified plastic surgeons, enhancing the reliability of content assessments.

However, several limitations must be acknowledged. First, the study examined a predefined set of questions, which may not fully reflect the diversity of real-world patient inquiries. Since LLM-generated responses are highly sensitive to prompt phrasing, variations in user input could yield different results, affecting reproducibility and generalizability. Second, our analysis was limited to a single LLM (ChatGPT-4), and findings may not extend to other models such as Claude or Gemini [52], which differ in training data, architectures, and response-generation methodologies. Third, while expert evaluation provided clinical insight into content quality, inter-rater reliability was not formally assessed, introducing potential subjectivity in scoring. Additionally, the study did not measure the actual impact of AI-generated information on patient comprehension or decision-making, an important factor for evaluating real-world applicability. Finally, ChatGPT does not provide citations or verifiable references, raising concerns about the reliability of unsupervised AI-generated health information.

## 5. Conclusions

This study highlights ChatGPT’s potential as a supplementary tool for patient education in cleft lip repair, demonstrating generally accurate, relevant, and understandable information. While language models can support patient education, they should complement, not replace, traditional clinician-led discussions. As these technologies evolve, their role in clinical practice will likely expand, enhancing patient engagement and understanding. Further studies are needed to explore the real-world impact of AI-assisted education on patient comprehension and decision-making in clinical settings.

## Figures and Tables

**Table 1:** Ten questions extracted from American Society of Plastic Surgeons patient information page for cleft lip repair.

1. What is cleft lip repair surgery?
2. What causes a cleft lip?
3. When should my child have cleft lip surgery?
4. How long is a cleft lip repair surgery?
5. How long of a recovery period can be expected from cleft lip repair surgery?
6. What kind of help will my child need during recovery after cleft lip repair surgery?
7. What are the risks and complications associated with cleft lip repair surgery?
8. What are my options if I am dissatisfied with the outcome of my child’s cleft surgery?
9. What team of specialists are involved in cleft lip repair?
10. How should I prepare for my child’s cleft lip repair?

**Table 2:** The definitions used for the 4-item Likert scale in the assessment.

Score	Overall	Content	Clarity	Relevance	Trustworthiness
4	Very satisfied	Completely correct	Exceptionally clear and easy to understand	Extremely relevant and directly applicable to clinical practice	Completely trustworthy as a reliable and accurate source
3	Satisfied	Correct but insufficient	Mostly clear with minimal ambiguity	Relevant and usefulclinical content	Trustworthy as a reliable source of information
2	Dissatisfied	A combination of correct and incorrect information	Clarity in some parts but was confusing in others	Some relevance but was not directly applicable	Somewhat trustworthy but would be cautious of this information
1	Very dissatisfied	Completely incorrect	Unclear and confusing content	Not relevant or applicable	Not trustworthy at all

## Data Availability

All data generated or analyzed during this study are included in this published article.

## Appendix A

ChatGPT Output for each question.

Response to Question 1: Cleft lip repair surgery is a surgical procedure to correct a cleft lip, which is a congenital condition where there is an opening or gap in the upper lip. This gap occurs during fetal development when the tissues that form the upper lip do not fully fuse. During the surgery, a plastic surgeon carefully stitches the tissues together to close the gap and create a more normal appearance. The goal is not only cosmetic but also functional, as cleft lip can affect feeding, speech, and facial development. The procedure is typically performed when the child is a few months old, and additional surgeries may be needed as the child grows to address any remaining issues. Cleft lip repair is often part of a comprehensive treatment plan that may also include cleft palate repair and ongoing care from a multidisciplinary team of healthcare professionals.

Response to Question 2: A cleft lip is typically caused by a combination of genetic and environmental factors. It occurs during early fetal development when the tissues that form the upper lip fail to fuse properly. The exact cause of this failure to fuse is not always clear, but several factors may contribute: (1) **Genetics:** There is often a genetic component to cleft lip, meaning it can run in families. Certain gene mutations or variations may increase the risk of a baby developing a cleft lip. (2) **Environmental factors:** Exposure to certain environmental factors during pregnancy can increase the risk of cleft lip. These factors may include maternal smoking, alcohol consumption, certain medications, and infections. (3) **Nutritional deficiencies:** A lack of certain nutrients during pregnancy, such as folic acid, may contribute to the development of cleft lip. (4) **Hormonal factors:** Changes in maternal hormones during pregnancy may play a role in the development of cleft lip. It’s important to note that in many cases, the exact cause of a cleft lip is not identified, and it may be a combination of genetic and environmental factors. Early diagnosis and intervention through surgical repair can help address the condition and minimize its impact on a child’s development.

Response to Question 3: Cleft lip surgery is typically performed when the child is a few months old. The exact timing may vary based on the specific circumstances of the child and the recommendations of the medical team. However, here are some general guidelines: (1) **Primary repair:** The initial surgery to repair the cleft lip is often done when the baby is between 3 to 6 months old. Performing the surgery at this age allows for early correction, which can have positive effects on the child’s feeding, speech development, and overall facial appearance. (2) **Health and weight considerations:** The child should be in good health and have reached a suitable weight for the surgery. This ensures that the child can tolerate the anesthesia and recover well from the procedure. (3) **Cleft palate repair timing:** If the child also has a cleft palate, additional surgeries to repair the palate may be needed. The timing of cleft palate repair is usually determined by the surgeon and may occur between 6 months to 18 months of age. (4) **Team approach:** The decision on the timing of cleft lip surgery is often made collaboratively by a team of healthcare professionals, including pediatricians, plastic surgeons, and speech therapists. Early intervention is crucial for addressing the functional and cosmetic aspects of cleft lip, and the healthcare team will provide guidance on the most appropriate timing for the child’s specific situation. Regular follow-up appointments are typically scheduled to monitor the child’s progress and address any ongoing needs.

Response to Question 4: The duration of cleft lip repair surgery can vary depending on the complexity of the cleft and the specific techniques used by the surgeon. On average, a cleft lip repair surgery typically takes about 1 to 2 h. During the surgery, the plastic surgeon carefully closes the gap in the upper lip, reconstructing the tissues to create a more normal appearance. The exact steps and techniques employed may vary, and in some cases, additional procedures may be performed simultaneously, such as addressing the nasal deformity or other related issues. While the surgery itself may be relatively short, the overall process involves preoperative preparation, anesthesia induction, and postoperative recovery. After the surgery, the child will be closely monitored in the recovery room before being moved to a hospital room for further observation. It’s important to note that the child’s age, overall health, and any additional procedures required can influence the duration of the surgical process. The healthcare team will provide specific information and guidance based on the individual circumstances of each case.

Response to Question 5: The recovery period after cleft lip repair surgery varies, but most children can expect a relatively quick recovery compared to other types of surgeries. Here are some general aspects of the recovery process: (1) **Immediate postoperative period:** The child will spend some time in the recovery room immediately after surgery, where medical staff will monitor vital signs and ensure a smooth transition from anesthesia. This period usually lasts a few hours. (2) **Hospital stay:** In many cases, children can go home on the same day as the surgery or the day after. The hospital stay is typically short, and the child may be discharged once they are eating well, managing pain adequately, and showing signs of stable recovery. (3) **Pain management:** Pain management is an essential part of the recovery process. The healthcare team will provide guidance on pain medications and other strategies to keep the child comfortable during the initial days after surgery. (4) **Follow-up appointments:** Regular follow-up appointments with the surgeon will be scheduled to monitor the healing process. These appointments help ensure that the surgical site is healing well and that there are no complications. (5) **Activity restrictions:** While the child may resume normal activities gradually, there may be some restrictions on certain activities to prevent injury to the surgical site. The surgeon will provide specific guidelines on when the child can return to regular activities. Overall, the recovery period can vary from a few days to a couple of weeks, and the child’s individual response to the surgery plays a role. It’s essential for parents to follow the postoperative care instructions provided by the healthcare team to promote optimal healing and minimize potential complications.

Response to Question 6: During the recovery period after cleft lip repair surgery, a child may need various types of support and care from parents and caregivers. Here are some aspects to consider: (1) **Pain management:** The child may experience some discomfort or pain after surgery. Administering prescribed pain medications as directed by the healthcare team is crucial to ensure the child’s comfort during the initial stages of recovery. (2) **Feeding assistance:** Depending on the age of the child, feeding may require some adjustments. Parents may need to use special feeding techniques or utensils to ensure the child can eat comfortably. It’s essential to follow any feeding guidelines provided by the healthcare team. (3) **Oral hygiene:** Proper oral hygiene is important during the recovery period. Parents may need to clean the surgical site gently and follow any specific instructions provided by the surgeon or dental care team. (4) **Monitoring for signs of complications:** Keep a close eye on the surgical site for any signs of infection, swelling, or unusual changes. Report any concerns to the healthcare team promptly. (5) **Emotional support:** The child may feel anxious or stressed during the recovery process. Providing emotional support, reassurance, and comfort can contribute to a smoother recovery. Encourage the child to express their feelings and communicate openly about the experience. (6) **Follow-up appointments:** Attend all scheduled follow-up appointments with the surgeon and healthcare team. These appointments are essential for monitoring the healing process and addressing any concerns that may arise. (7) **Activity restrictions:** Follow any activity restrictions or guidelines provided by the healthcare team to prevent injury to the surgical site. Gradually reintroduce regular activities as recommended by the surgeon. It’s important to work closely with the healthcare team to ensure that the child’s recovery is progressing well. Clear communication, adherence to postoperative care instructions, and a supportive environment at home contribute to a positive and successful recovery after cleft lip repair surgery.

Response to Question 7: While cleft lip repair surgery is generally considered safe and routine, like any surgical procedure, it carries some risks and potential complications. It’s important for parents and caregivers to be aware of these possibilities and discuss them with the healthcare team. Some potential risks and complications include: (1) **Infection:** There is a risk of infection at the surgical site. It’s important to keep the area clean and follow the postoperative care instructions to minimize this risk. (2) **Bleeding:** Some bleeding is normal after surgery, but excessive bleeding can occur. It’s crucial to monitor for any signs of increased bleeding and seek prompt medical attention if necessary. (3) **Scarring:** While plastic surgeons aim to minimize scarring, some scarring is inevitable. The appearance of the scar can vary, and additional procedures may be considered later for scar revision if needed. (4) **Anesthesia risks:** Anesthesia always carries some inherent risks. The anesthesia team will carefully assess the child’s health and take measures to minimize these risks. (5) **Asymmetry:** Achieving perfect symmetry in the repaired lip may be challenging, and there may be some degree of asymmetry in the final result. (6) **Nasal issues:** Cleft lip repair may also affect the nasal structures, and there could be changes in nasal appearance or function. Additional procedures may be necessary to address these issues. (7) **Feeding difficulties:** While cleft lip repair aims to improve feeding, some children may still experience difficulties. Special feeding techniques or devices may be required. (8) **Speech concerns:** Cleft lip repair may influence speech development, and some children may require speech therapy to address any related issues. It’s important to note that serious complications are rare, and the majority of children experience successful outcomes from cleft lip repair surgery. The healthcare team will discuss potential risks and complications with parents before the surgery, and close monitoring during the recovery period helps address any issues promptly. Regular follow-up appointments allow the healthcare team to assess the child’s progress and address any concerns that may arise.

Response to Question 8: If you are dissatisfied with the outcome of your child’s cleft surgery, it’s important to address your concerns and explore your options for further evaluation and potential corrective procedures. Here are steps you can take: (1) Communicate with the Surgical Team: The first and most crucial step is to communicate your concerns with the surgical team that performed the initial cleft surgery. They need to be aware of your dissatisfaction so they can assess the situation and discuss potential solutions. Schedule an appointment to meet with the surgeon and the healthcare professionals involved in your child’s care. Share your specific concerns and questions about the surgical outcome. (2) Seek a Second Opinion: If you remain dissatisfied or have reservations about the initial surgical outcome, consider seeking a second opinion from another experienced cleft and craniofacial surgeon. A fresh perspective can provide valuable insights and recommendations. (3) Comprehensive Evaluation: A comprehensive evaluation of your child’s condition may be necessary. This could involve additional imaging, such as X-rays or scans, to assess the underlying structures and tissues. Speech and language evaluations may also be conducted to assess any speech issues related to the cleft. (4) Revision Surgery: In some cases, it may be appropriate to consider revision surgery to address specific concerns or improve the outcome. Revision surgery is a surgical procedure performed after the initial surgery to make corrections or improvements. Revision surgery is carefully planned and discussed with the surgical team to ensure that the best possible outcome is achieved. (5) Speech Therapy and Rehabilitation: If your child is experiencing speech or functional issues related to the cleft repair, speech therapy and rehabilitation services can be beneficial. These therapies can help improve speech and overall oral function. (6) Emotional Support: It’s important to provide emotional support for your child throughout this process, as they may have their own feelings and concerns about the surgical outcome. Consider seeking counseling or support groups for both you and your child to help cope with any emotional challenges. (7) Review Insurance and Healthcare Coverage: Review your health insurance coverage to understand the extent to which revision surgery and related therapies may be covered. Consult with your insurance provider to clarify coverage and financial aspects. Remember that the goal of addressing concerns and seeking further evaluation or revision surgery is to ensure the best possible outcome and quality of life for your child. It’s crucial to work collaboratively with a team of experienced healthcare professionals who specialize in cleft and craniofacial care to address your child’s unique needs. Open communication, thorough evaluation, and careful planning are essential in these situations.

Response to Question 9: Cleft lip repair typically involves a multidisciplinary team of specialists who work together to provide comprehensive care for the child. The team may include: (1) **Plastic Surgeon:** A plastic surgeon with expertise in cleft lip and palate repair performs the surgical procedure. The surgeon focuses on achieving both functional and cosmetic improvements. (2) **Pediatrician:** A pediatrician plays a key role in the overall health and well-being of the child. They may be involved in the preoperative assessment, coordination of care, and ongoing monitoring of the child’s development. (3) **Orthodontist:** An orthodontist may be involved in the long-term management of the child’s oral and facial development. They may address issues such as dental alignment, jaw growth, and the coordination of orthodontic care with other treatments. (4) **Speech Therapist:** Speech therapists help address any speech or language issues that may arise due to the cleft lip and palate. They work with the child to improve communication skills and assist in overcoming potential speech challenges. (5) **Ear, Nose, and Throat (ENT) Specialist:** ENT specialists may be involved in addressing issues related to the ear and nose, which can be affected by cleft lip and palate. They may assist in managing ear infections, hearing issues, and nasal concerns. (6) **Genetic Counselor:** A genetic counselor may work with the family to provide information about the genetic aspects of cleft lip and palate, including any potential recurrence risks in future pregnancies. (7) **Nutritionist:** A nutritionist may be involved in providing guidance on feeding techniques, nutritional needs, and monitoring the child’s growth and development. (8) **Social Worker or Psychologist:** Emotional and psychological support is crucial for both the child and the family. Social workers or psychologists can assist in addressing any emotional or psychosocial challenges that may arise. (9) **Dental Specialist:** Depending on the specific needs of the child, a dental specialist may be involved in addressing dental issues related to cleft lip and palate. This collaborative approach ensures that the child receives comprehensive care that addresses the various aspects of cleft lip and palate, including surgical, medical, dental, speech, and psychosocial needs. Regular communication and coordination among team members help provide the best possible outcomes for the child.

Response to Question 10: Preparing for your child’s cleft lip repair involves a combination of practical and emotional considerations. Here are some general guidelines to help you prepare: (1) **Educate yourself:** Learn as much as you can about the cleft lip repair procedure, the recovery process, and potential challenges. This knowledge will help you make informed decisions and better support your child. (2) **Communicate with the healthcare team:** Establish open communication with the healthcare team, including the plastic surgeon and other specialists involved. Discuss any concerns, ask questions, and ensure that you understand the details of the procedure, recovery plan, and long-term care. (3) **Coordinate logistics:** Plan for the logistics of the surgery, such as transportation to and from the hospital, accommodation if needed, and any necessary time off from work. Having these details sorted out in advance can reduce stress on the day of the surgery. (4) **Arrange for support:** Enlist the support of family members, friends, or a support network to help you during the recovery period. Having assistance with daily tasks can be invaluable. (5) **Prepare your home:** Create a comfortable and supportive environment for your child’s recovery. Set up a quiet and restful space, have necessary supplies on hand, and ensure that you have any recommended feeding or care tools.

(6) **Follow preoperative instructions:** The healthcare team will provide specific preoperative instructions. This may include guidelines on fasting before the surgery, medication adjustments, and other preparations. Adhering to these instructions is crucial for a smooth surgical experience. (7) **Emotional preparation:** Acknowledge and address your own emotions and concerns. Seek support from friends, family, or counseling services if needed. Taking care of your emotional well-being allows you to better support your child. (8) **Pack a hospital bag:** If your child will stay overnight at the hospital, pack a bag with essentials such as comfortable clothing, toiletries, and any comfort items for your child. (9) **Learn about postoperative care:** Familiarize yourself with the postoperative care instructions, including medication administration, feeding guidelines, and signs of complications. This knowledge will empower you to provide optimal care for your child during the recovery period. Remember that each child’s experience is unique, and the healthcare team is there to guide and support you. By being proactive and well-prepared, you can contribute to a positive experience for both you and your child during the cleft lip repair journey.
